# Tuina (Chinese massage) for insulin resistance and sensitivity: A protocol for systematic review and meta-analysis of animal and human studies

**DOI:** 10.1371/journal.pone.0288414

**Published:** 2023-07-20

**Authors:** Zhixuan Zhao, Jun Yan, Yuxin Ding, Yingji Wang, Yan Li

**Affiliations:** 1 Department of Integrated Chinese and Western Medicine, Hongqi Hospital of Mudanjiang Medical University, Mudanjiang, 157011, China; 2 Department of Oncology, First Affiliated Hospital, Heilongjiang University of Chinese Medicine, Harbin, 150040, China; 3 Department of Tuina, First Affiliated Hospital, Heilongjiang University of Chinese Medicine, Harbin, 150040, China; 4 College of Pharmacy, Harbin Medical University, Harbin, 150081, China; 5 TCM Translational Medicine Research Center, First Affiliated Hospital, Heilongjiang University of Chinese Medicine, Harbin, 150040, China; UFRN: Universidade Federal do Rio Grande do Norte, BRAZIL

## Abstract

**Introduction:**

Insulin resistance (IR) could be regarded as a therapeutic target for metabolic diseases. Therefore, multiple therapeutic strategies that target IR should be applied to provide a more effective means of treatment. It aims to determine Tuina’s efficacy and safety for IR through this systematic review and meta-analysis.

**Methods:**

From the inception to July 31, 2023, we will search four English databases (Pubmed, Embase, Cochrane Central Register of Controlled Trials, Web of Science) and two Chinese databases (China National Knowledge Infrastructure and the Chinese Science and Technology Periodical Database). We will search and include studies of both human and animal models that evaluate Tuina’s effects on insulin sensitivity or resistance. Data selection, data extraction, and risk of bias assessment will be made by two independent reviewers. We will evaluate the methodological quality of all included studies and conduct meta-analyses using Review Manager Software 5.4.1.

**Discussion:**

In both animal and human studies, the effects and safety of Tuina for IR will be evaluated. The evidence generated bythis study will validate effects and safety of Tuinain treating IR and inform future research and clinical decision-making.

**Trail registration:**

PROSPERO Registration ID: CRD42022360128.

## Introduction

As a commonly seen pathological condition, insulin resistance (IR) is primarily associated with resistance to insulin-mediated glucose disposal [1]. In recent years, it has been demonstrated that IR is associated with a broad spectrum of chronic morbidities, including obesity, metabolic syndrome (MetS), type 2 diabetes mellitus (T2DM), polycystic ovarian syndrome (PCOS), and cardiovascular diseases [2,3].

There is a close connection between obesity and IR, a complex interplay of genetic and environmental factors seems to be involved in obesity-induced insulin resistance [4], and the pandemic of obesity caused by modern lifestyles has increased the prevalence of IR [5,6]. In the U.S., the incidence of IR increased by 35.1% in nondiabetic adult [7]; in obese children and adolescence, the incidence of IR reaches 47.1% to 52.1% [8,9]. In developing countries, notably in Asia, there is a rapid increase in diseases associated with IR [10].

MetS, also known as insulin resistance syndrome, consists of a group of metabolic abnormalities, including glucose metabolism disorder, hypertension, dyslipidaemia and obesity [11,12] and it is believed that IR is responsible for the occurrence of MetS components [13]. In the circumstances of IR, target tissues including skeletal muscle, liver, and adipose tissue cannot provide an expected response under normal insulin level; therefore, pancreatic beta-cells secrete more insulin to overcome hyperglycaemia [14]. When pancreatic beta-cells cannot secrete enough insulin to overcome IR, T2DM manifests [14].

Althoughthe exact etiology remains incompletely comprehended, IR is widely acknowledged as an important underlying mechanism associated with T2DM [15]; and in T2DM, IR disrupts glycaemic homeostasis, causing pancreatic beta cell failure [16]. Overproduction of cytokines and reactive oxygen species could contribute to IR, which could be associated with MetS [17].

Even though not included in the diagnostic criteria, IR is a very common condition in PCOS patients. It is reported that the incidence of IR is 59.3% in normal-weight subjects, 77.5% in overweight subjects, and 93.9% in obese subjects [18]. However, the molecular mechanisms of IR in PCOS seem to vary from other disorders such as obesity and T2DM [19].

Despite the fact that numerous researchers are studying IR, there is little underlying knowledge about its origins and development. Various mechanisms have been proposed, including peripheral IR and defective insulin secretion [20].

As one of the common causes of MetS, IR should be considered a therapeutic target for metabolic diseases, including diabetes [21,22]. Thus, attenuating IR has always been the foremost therapeutic goal. Therapeutic strategies for IR include changing dietary-lifestyle habits, applying pharmaceutical and surgical approaches, and complementary and alternative medicine therapies [23–27].

Chinese massage (also known as Tuina) is a traditional form of physical therapy in Chinese medicine that traces its origins to 220 BC. Tuina follows the theory of Chinese medicine: the practitioner may brush, knead, roll, press, and rub the areas [28]. From the perspective of modern medicine, Tuina can dilate blood vessels, promote blood flow, improve microcirculation [29], promote and improve insulin secretion [30–32], improve the regulation function of the central nervous system [33,34] and autonomic nervous system [35], boostimmunity [36,37], strengthen metabolism in the body [38], and ensure thatglucose in muscle tissue is fully utilized to achieve the purpose of lowering blood sugar and treating diabetes [39,40].

The potential effects of Tuina on IR have been studied previously in both human [41,42] and animal studies [39,40], and its benefits have been reported, but the efficacy of Tuina in treating IR arouses controversy. Therefore, the purpose of the present study is to evaluate the effectiveness and safety of Tuina in treating IR.

## Methods

### Study registration

This systematic review was prospectively registered on PROSPERO (CRD42022360128).This protocol is reported according to the guidelines of the preferred reporting items for systematic review and meta-analysis protocols (PRISMA-P) [43] and is followed the structure provided in the Systematic Review Protocol for Animal Intervention Studies [44].

### Inclusion criteria

#### Types of studies

This study will include both randomized controlled trial (RCT) and non-randomized controlled trial (NRCT) published in English and Chinese. There will be no restriction on dates.

#### Types of patients and animal models

We will include studies of both human and animal models that evaluate Tuina’s effects on insulin sensitivity or resistance. No restrictions will be placed on the patients’ age, gender, location, race, disease, and the course of the disease. Models of all animals will be included, regardless of their species or size.

#### Types of interventions and comparator(s)/control

This study will include Tuina, guided by Chinese medicine theory, without restrictions on treatment types or duration. The comparators will consist of no treatment, sham Tuina, placebo Tuina point control, and other active interventions, including medications, exercise, and other complementary and alternative medicines. We will also include studies comparing Tuina in combination with another intervention to the same other intervention alone.

#### Types of outcomes

The primary outcome is the homeostasis model assessment of insulin resistance (HOMA-IR) since it is the most commonly used to assess IR in clinical practice [45].

Other outcomes include GDR (glucose disposal rate) evaluated by hyperinsulinemic-euglycemic clamp, fasting plasma glucose (FBG), fasting insulin (FINS), glucose tolerance test (GTT), insulin challenge test (ICT), body weight (BW), body mass index (BMI), other insulin resistance surrogate indices such as QUICKI (quantitative insulin sensitivity check index) and adverse events.

### Collection and analysis of data

#### Search strategy

Pubmed, Embase, Cochrane Central Register of Controlled Trials, Web of Science, China National Knowledge Infrastructure (CNKI), and the Chinese Science and Technology Periodical Database (VIP) will be searched from inception to July 31, 2023. A detailed search strategy is shown in Table 1.

**Table 1:** Search strategy for Pubmed.

1	Insulin resistance/
2	Exp Resistance Insulin/
3	Exp Resistance Sensitivity/
4	Exp Sesitivity, Resistance/
5	Exp Insulin
6	OR/ 1–5
7	Tuina
8	Massage/
9	Exp Zone Therapy
10	Exp Therapies, Zone
11	Exp Zone Therapies
12	Exp Therapy, Zone
13	Exp Massage Therapy
14	Exp Massage Therapies
15	Exp Therapies, Massage
16	Exp Therapy, Massage
17	OR/ 8–16
18	Randomized controlled trial/
19	randomized.tw
20	randomly.tw
21	trial.tw
22	OR/ 18–21
23	Animal
24	22 OR 23
25	6 AND 17 AND 24

#### Data selection

**Fig 1:**
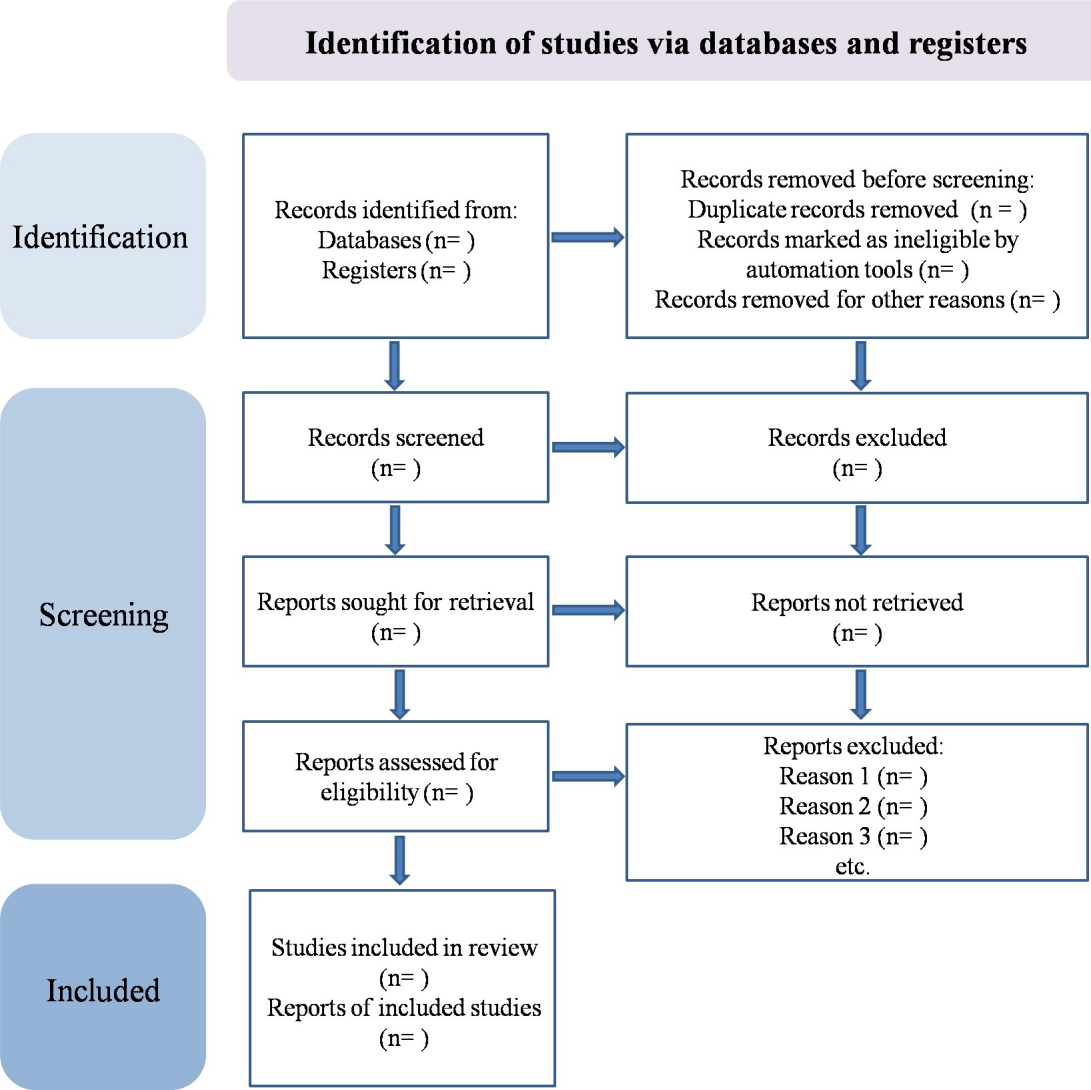
Flow chart of article screening and selection process.

We will enter the results of all searches into one single EndNote library, and there will be an identification and removal of duplicate studies. Two review authors (ZZ and JY) will review the titles and abstracts of each study independently, and the third review author (YD) will assist in resolving any disagreements. We will request additional information from the authors of the studies if necessary. PRISMA flow chart (Fig 1) illustrates the screening process.

#### Data extraction

A standardized spreadsheet will be created before data extraction. Two qualified authors (YW and YL) will extract data from all included studies and fillout the data extraction form. We will extract the following information: (1) features of the study, such as location, setting, study design, sample size, duration, funding sources, and objectives study characteristics, including location, setting, study design, size, duration, and study objectives; (2) features of human subjects,including age, sex, BMI,condition, duration, activity and exercise status, and dietary habits; (3) features of animal models, including species, age, body mass, source, sex, genotype, typeof condition, and modeling method; (4) interventions for treatment and control, such as the type of Tuina method used, the type of control used, the duration, frequency, medication administration, and dosage; (5) outcome characteristics for IR, including the type of measurement, sample sizes, data at baseline and end-of-treatment, intervention adherence, dropout rates and reasons, and the number and nature of adverse events. We will extract measures of central tendency and dispersion from figures in the articles using Web Plot Digitizer 4.5 (https://apps.automeris.io/wpd/), if necessary. If there is insufficient or ambiguous data, an email requesting additional information or clarification will be sent to the authors of the original studies. A third reviewer (YD) will resolve any disagreements raised.

#### Risk of bias assessment

In human trials, we will assess the risk of bias using the Cochrane Risk of Bias 2.0 (RoB 2.0) tool [46] for RCTs and the Risk Of Bias In Non-randomized Studies of Interventions (ROBINS-I) tool [47] for NRCTs. We will assess the risk of bias in animal studies using the Systematic Review Centre for Laboratory Animal Experimentation (SYRCLE) tool [48]. Biases will be classified as low risk, high risk, or some concerns as described in ROB 2.0.Accordingto the ROBINS-I, the overall risk of bias judgment for the outcome being assessed will be low risk, moderate risk, serious risk, critical risk, and no information. Based on SYRCLE statement, the studies will be classified as low bias, high bias, or unclear bias. Two authors (ZZ and YL) will assess the risk of bias independently, and any disagreements will be discussed further by all authors.

### Statistical analysis

#### Assessment of heterogeneity

To assess heterogeneity in the included studies, forest plots will be inspected visually. Cochran’s Q and I^2^ tests will be used to assess statistical heterogeneity across studies. I^2^> 50% indicates statistically significant heterogeneity exist, and the overall treatment effect will be determined by a meta-analysis using a random-effects model. If the I^2^ is<50%, a fixed-effects model will be used. In case of heterogeneity, a subgroup analysis will be conducted.

#### Synthesis of data

Detailed information about the study subjects, interventions, and outcomes of the studies will be presented in the form of summary tables and descriptive text in the review.We will conduct meta-analyses using Review Manager Software 5.4.1. To determine treatment effects, we will calculate the risk ratio (RR) with 95% confidence intervals (CI) for dichotomous outcomes and the mean difference (MD) with a 95% CI for continuous data.A standardized mean differences (SMDs) analysis with a 95% CI will be performed if the outcomes are measured on different scales.

#### Sensitivity analysis

Sensitivity analysis will be performed based on the overall risk of bias if there has been sufficient research for each intervention and outcome. We will recalculate the estimates, excluding studies with a high or unclear risk of bias in specific domains.

#### Subgroup analysis

We will perform a subgroup analysis to identify reasons for heterogeneity according to variations in the treatment, disease/condition and controls.

#### Publication bias

We will generate a funnel plot to evaluate potential publication bias.

#### Quality of evidence

**Fig 2:**
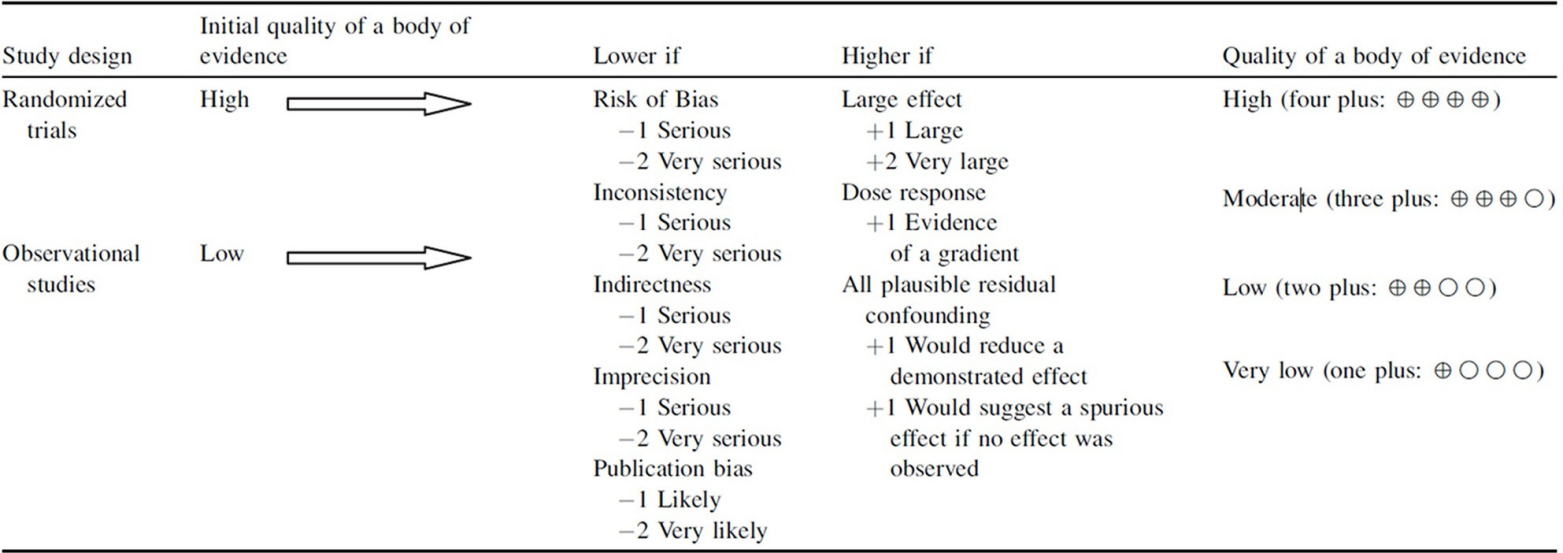
Rating the quality of evidence of GRADE’s approach [50].

Each outcome will be evaluated using GRADE (Grading of Recommendations Assessment, Development and Evaluation) [49]. Rating the quality of the evidence will begin with study design, there are five domains can downgrade the quality and three domains can upgrade the quality (Fig 2) [50]. Two independent investigators (ZZ and YL) will independently evaluatethe quality of the evidence. The quality will be graded as “high,” “moderate,” “low,” or “very low”.

## Discussion

In this study, we will comprehensively review the studies of Tuina on IR, including both human and animal studies. It will help guide future research and clinical decisions by generating evidence for the efficacy and safety of Tuina in treating IR.

## Supporting information

S1 Checklist PRISMA-P (Preferred Reporting Items for Systematic review and Meta-Analysis Protocols) 2015 checklist: Recommended items to address in a systematic review protocol*. (DOC)

## References

[1] Lebovitz HE. Insulin resistance: definition and consequences. Exp Clin Endocrinol Diabetes. 2001;109 Suppl 2:S135–148. doi: 10.1055/s-2001-18576 11460565

[2] Hill MA, Yang Y, Zhang L, Sun Z, Jia G, Parrish AR, et al. Insulin resistance, cardiovascular stiffening and cardiovascular disease. Metabolism. 2021; 119:154766. doi: 10.1016/j.metabol.2021.154766 33766485

[3] Lee SH, Park SY, Choi CS. Insulin Resistance: From Mechanisms to Therapeutic Strategies. Diabetes Metab J 2022;46(1):15–37. doi: 10.4093/dmj.2021.0280 34965646 PMC8831809

[4] Samuel VT, Shulman GI. Mechanisms for insulin resistance: common threads and missing links. Cell. 2012;148(5):852–871. doi: 10.1016/j.cell.2012.02.017 22385956 PMC3294420

[5] Finucane MM, Stevens GA, Cowan MJ, Danaei G, Lin JK, Paciorek CJ, et al. Global Burden of Metabolic Risk Factors of Chronic Diseases Collaborating Group (Body Mass Index). National, regional, and global trends in body-mass index since 1980: systematic analysis of health examination surveys and epidemiological studies with 960 country-years and 9·1 million participants. Lancet. 2011;377(9765):557–567. 21295846 10.1016/S0140-6736(10)62037-5 PMC4472365

[6] Goh LPW, Sani SA, Sabullah MK, Gansau JA. The Prevalence of Insulin Resistance in Malaysia and Indonesia: An Updated Systematic Review and Meta-Analysis. Medicina (Kaunas). 2022;58(6):826. doi: 10.3390/medicina58060826 35744089 PMC9227905

[7] Li C, Ford ES, McGuire LC, Mokdad AH, Little RR, Reaven GM. Trends in hyperinsulinemia among nondiabetic adults in the U.S. Diabetes Care. 2006;29(11):2396–2402. doi: 10.2337/dc06-0289 17065674

[8] Yi KH, Hwang JS, Kim EY, Lee SH, Kim DH, Lim JS. Prevalence of insulin resistance and cardiometabolic risk in Korean children and adolescents: a population-based study. Diabetes Res Clin Pract. 2014;103(1):106–113. doi: 10.1016/j.diabres.2013.10.021 24290751

[9] Lee JM, Okumura MJ, Davis MM, Herman WH, Gurney JG. Prevalence and determinants of insulin resistance among U.S. adolescents: a population-based study. Diabetes Care. 2006;29(11):2427–2432. doi: 10.2337/dc06-0709 17065679

[10] Hu FB. Globalization of diabetes: the role of diet, lifestyle, and genes. Diabetes Care. 2011;34(6):1249–1257. doi: 10.2337/dc11-0442 21617109 PMC3114340

[11] Alberti KG, Zimmet PZ. Definition, diagnosis and classification of diabetes mellitus and its complications. Part 1: diagnosis and classification of diabetes mellitus provisional report of a WHO consultation. Diabet Med. 1998;15(7):539–553. doi: 10.1002/(SICI)1096-9136(199807)15:7&lt;539::AID-DIA668&gt;3.0.CO;2-S 9686693

[12] Alberti KG, Eckel RH, Grundy SM, Zimmet PZ, Cleeman JI, Donato KA, et al. International Diabetes Federation Task Force on Epidemiology and Prevention; Hational Heart, Lung, and Blood Institute; American Heart Association; World Heart Federation; International Atherosclerosis Society; International Association for the Study of Obesity. Harmonizing the metabolic syndrome: a joint interim statement of the International Diabetes Federation Task Force on Epidemiology and Prevention; National Heart, Lung, and Blood Institute; American Heart Association; World Heart Federation; International Atherosclerosis Society; and International Association for the Study of Obesity. Circulation. 2009;120(16):1640–1645. doi: 10.1161/CIRCULATIONAHA.109.192644 19805654

[13] DeFronzo RA, Ferrannini E. Insulin resistance. A multifaceted syndrome responsible for NIDDM, obesity, hypertension, dyslipidemia, and atherosclerotic cardiovascular disease. Diabetes Care. 1991;14(3):173–194. doi: 10.2337/diacare.14.3.173 2044434

[14] Kahn SE, Hull RL, Utzschneider KM. Mechanisms linking obesity to insulin resistance and type 2 diabetes. Nature. 2006;444(7121):840–846. doi: 10.1038/nature05482 17167471

[15] Nolan CJ, Prentki M. Insulin resistance and insulin hypersecretion in the metabolic syndrome and type 2 diabetes: Time for a conceptual framework shift. Diab Vasc Dis Res. 2019;16(2):118–127. doi: 10.1177/1479164119827611 30770030

[16] Thomas CC, Philipson LH. Update on diabetes classification. Med Clin North Am. 2015;99(1):1–16. doi: 10.1016/j.mcna.2014.08.015 25456640

[17] Wittmann I. Insulin Resistance and Metabolic Syndrome. EJIFCC. 2007;18(1):31–38. 29632465 PMC5875079

[18] Tosi F, Bonora E, Moghetti P. Insulin resistance in a large cohort of women with polycystic ovary syndrome: a comparison between euglycaemic-hyperinsulinaemic clamp and surrogate indexes. Hum Reprod. 2017;32(12):2515–2521. doi: 10.1093/humrep/dex308 29040529

[19] Moghetti P, Tosi F. Insulin resistance and PCOS: chicken or egg? J Endocrinol Invest. 2021;44(2):233–244. doi: 10.1007/s40618-020-01351-0 32648001

[20] Yaribeygi H, Farrokhi FR, Butler AE, Sahebkar A. Insulin resistance: Review of the underlying molecular mechanisms. J Cell Physiol. 2019;234(6):8152–8161. doi: 10.1002/jcp.27603 30317615

[21] Rosenberg DE, Jabbour SA, Goldstein BJ. Insulin resistance, diabetes and cardiovascular risk: approaches to treatment. Diabetes ObesMetab. 2005;7(6):642–653. doi: 10.1111/j.1463-1326.2004.00446.x 16219008

[22] Matthaei S, Stumvoll M, Kellerer M, Häring HU. Pathophysiology and pharmacological treatment of insulin resistance. Endocr Rev. 2000;21(6):585–618. doi: 10.1210/edrv.21.6.0413 11133066

[23] Yang J, Liu Y, Huang J, Xu J, You X, Lin Q, et al. [Acupuncture and Chinese medicine of artificial cycle therapy for insulin resistance of polycystic ovary syndrome with phlegm damp type and its mechanism]. Zhongguo Zhen Jiu. 2017;37(11):1163–1168. doi: 10.13703/j.0255-2930.2017.11.007 29354951

[24] Rogowicz-Frontczak A, Majchrzak A, Zozulińska-Ziółkiewicz D. Insulin resistance in endocrine disorders—treatment options. Endokrynol Pol. 2017;68(3):334–351. doi: 10.5603/EP.2017.0026 28660991

[25] Zhang Z, Leng Y, Chen Z, Fu X, Liang Q, Peng X, et al.The efficacy and safety of Chinese herbal medicine as an add-on therapy for type 2 diabetes mellitus patients with carotid atherosclerosis: An updated meta-analysis of 27 randomized controlled trials. Front Pharmacol. 2023; 14:1091718. doi: 10.3389/fphar.2023.1091718 37033624 PMC10076753

[26] Cordero P, Li J, Oben JA. Bariatric surgery as a treatment for metabolic syndrome. J R Coll Physicians Edinb. 2017;47(4):364–368. doi: 10.4997/JRCPE.2017.414 29537411

[27] Liu Y, Fan HY, Hu JQ, Wu TY, Chen J. Effectiveness and safety of acupuncture for insulin resistance in women with polycystic ovary syndrome: A systematic review and meta-analysis. Heliyon. 2023;9(3):e13991. doi: 10.1016/j.heliyon.2023.e13991 36923858 PMC10009463

[28] Yu TY. Science of Chinese massage. Beijing: Chinese Medicine Press; 2013.

[29] Xu SX, Ji L.Wang QW. Numerical Investigation of Effect of Rolling Manipulation of Traditional Chinese Medical Massage on Blood Flow. Applied Mathematics and Mechanics. 2005; 6:694–700.

[30] Zhang J, Ma DJ, Li HL, Wen YH. Clinical study of manipulation in the treatment of early type 2 diabetes mellitus. China Medical Herald.2013;10(16):115–117.

[31] Wang XB, Yang XY, Wang ZH. Clinical Study of the Tuina Therapy Guided by Dredging Meridians and Regulating Internal Organs for T2DM with Phlegm- dampness Stagnation Pattern. JCAM. 2017;33(06):36–38.

[32] Zhang X, Liu MJ, Wu XQ, Chen ST, Zhong CW, Fu YN, et al. Experimental Study on Effect of Abdomen- Activating and Meridians-Dredging Tuina Therapy on SIRT1/PGC-1α Pathway Protein and mRNA Expression in Skeletal Muscle of Obese Rats. Chinese archives of Traditional Chinese Medicine. 2020;38(11):22–25.

[33] Yuan WA, Cheng YW, Zhan HS. Research progress in influences of spinal manipulation on central nervous system. Academic Journal of Shanghai University of Traditional Chinese Medicine.2011;25(03):104–107.

[34] Li HN, Ma YL, Zhang W, Liu SW, Luo XF, Dong Y, et al. Mechanism of abdominal massage treating central nervous system diseases based on brain-gut axis theory. Liaoning Journal of Traditional Chinese Medicine.2019;46(11):2321–2324.

[35] Cui KM, Li WM, Liu X, Budgell B, Li N, Wu GC. The influence of cervical spine massage therapy on autonomic in nerve function in healthy volunteers. Shanghai Journal of Acupuncture and Moxibustion.2006; 06:6–8.

[36] Guo GX, Zhu GQ, Sun WQ, Kong LJ, Xu SD, Zhou Xin, et al. Research progress of immunological mechanism of tuina intervention on lumbardisc herniation. China Journal of Traditional Chinese Medicine and Pharmacy.2019;34(07):3132–3135.

[37] Wang ZH, Yang JY, Liu MJ, Zhuo Y, Yang JY, Yu MC, et al.Effect of back Tuinamanipulation on serum immune cells andimmunoglobulin in rabbits with subacute aging and immune dysfunction. Journal of Basic Chinese Medicine. 2020;26(05):622–624.

[38] Zhu TG, Han J, Shu JW, Ke DF, Wang D, Liu WJ, et al. In vivo breath analysis by extractive Electrospray Ionization-Mass Spectrometry for investigation of metabolic responsesto Traditional Chinese Medicine massages. Chinese Journal of Analytical Chemistry.2018;46(03):400–405.

[39] Han YR, Yan MH, Liu HH, Zhang XL, Liu MJ. Effects of the promoting channel abdominal massage on regulating insulinresistance in rats by SIRT1. China Journal of Traditional Chinese Medicine and Pharmacy. 2020;35(05):2568–2571.

[40] Meng M, Hu GY, Wu XQ, Cong DY. Effects of skeletal muscle massage on skeletal muscle function and conversion of skeletal muscle fiber types in type 2 diabetic rats. Chinese Journal of Tissue Engineering Research. 2023; 3:1–6.

[41] Wang Y. Effect analysis of massage on insulin resistance of simple obese children. Smart healthcare. 2023,35(01):155–160.

[42] Wang XB, Yang XY, Wang ZH. Clinical Study of the Tuina Therapy Guided by Dredging Meridians and Regulating Internal Organs for T2DM with Phlegm- dampness Stagnation Pattern. JCAM.2017;33(06):36–38.

[43] Shamseer L, Moher D, Clarke M, Ghersi D, Liberati A, Petticrew M, et al. PRISMA-P Group. Preferred reporting items for systematic review and meta-analysis protocols (PRISMA-P) 2015: elaboration and explanation. BMJ. 2015;350:g7647. doi: 10.1136/bmj.g7647 25555855

[44] De Vries RBM, Hooijmans CR, Langendam MW, van Luijk J., Leenaars M., Ritskes-Hoitinga M. et al. A protocol format for the preparation, registration and publication of systematic reviews of animal intervention studies. Evid-Based Preclinical Med. 2015; 2:1–9.

[45] Matthews DR, Hosker JP, Rudenski AS, Naylor BA, Treacher DF, Turner RC. Homeostasis model assessment: insulin resistance and beta-cell function from fasting plasma glucose and insulin concentrations in man. Diabetologia. 1985;28(7):412–419. doi: 10.1007/BF00280883 3899825

[46] Sterne JAC, Savović J, Page MJ, Elbers RG, Blencowe NS, Boutron I, et al.RoB 2: a revised tool for assessing risk of bias in randomised trials. BMJ. 2019;366: l4898. doi: 10.1136/bmj.l4898 31462531

[47] Sterne JAC, Hernán MA, Reeves BC, Savović J, Berkman ND, Viswanathan M, et al. ROBINS-I: a tool for assessing risk of bias in non-randomised studies of interventions. BMJ. 2016;355: i4919. doi: 10.1136/bmj.i4919 27733354 PMC5062054

[48] Hooijmans CR, Rovers MM, de Vries RB, Leenaars M, Ritskes-Hoitinga M, Langendam MW. SYRCLE’s risk of bias tool for animal studies. BMC Med Res Methodol. 2014; 14:43. doi: 10.1186/1471-2288-14-43 24667063 PMC4230647

[49] Guyatt G, Oxman AD, Akl EA, Kunz R, Vist G, Brozek J, et al. GRADE guidelines: 1. Introduction-GRADE evidence profiles and summary of findings tables. J Clin Epidemiol. 2011;64(4):383–394. doi: 10.1016/j.jclinepi.2010.04.026 21195583

[50] Balshem H, Helfand M, Schünemann HJ, Oxman AD, Kunz R, Brozek J, et al.GRADE guidelines: 3. Rating the quality of evidence. J Clin Epidemiol. 2011;64(4):401–406. doi: 10.1016/j.jclinepi.2010.07.015 21208779

